# Multiple Myeloma Immunophenotype Related to Chromosomal Abnormalities Used in Risk Assessment

**DOI:** 10.3390/diagnostics12092049

**Published:** 2022-08-24

**Authors:** Mantas Radzevičius, Vaidas Dirsė, Indrė Klimienė, Rėda Matuzevičienė, Zita Aušrelė Kučinskienė, Valdas Pečeliūnas

**Affiliations:** 1 Department of Physiology, Biochemistry, Microbiology and Laboratory Medicine, Institute of Biomedical Sciences, Faculty of Medicine, Vilnius University, 03101 Vilnius, Lithuania; 2 Hematology, Oncology and Transfusion Medicine Center, Vilnius University Hospital Santaros Klinikos, 08661 Vilnius, Lithuania; 3 Center of Laboratory Medicine, Vilnius University Hospital Santaros Klinikos, 08661 Vilnius, Lithuania; 4 Clinic of Internal Diseases, Family Medicine and Oncology, Institute of Clinical Medicine, Faculty of Medicine, Vilnius University, 03101 Vilnius, Lithuania

**Keywords:** flow cytometry, single nucleotide polymorphism microarray, multiple myeloma, immunophenotype, risk assessment

## Abstract

(1) Background: At diagnosis, multiplemyeloma risk estimation includes disease burden, end-organ damage, and biomarkers, with increasing emphasis on genetic abnormalities. Multicolor flow cytometry (MFC) is not always considered in risk estimation. We demonstrate associations found between genetic abnormalities and antigen expression of plasma cells measured by MFC. (2) Methods: Single nucleotide polymorphism microarray (SNP-A) karyotyping as well as MFC using standardized next-generation flow (NGF) panels and instrument settings were performed from bone marrow aspirates at the time of diagnosis. (3) Results: We uncovered specific immunophenotype features related to different genetic risk factors. Specifically, we found higher malignant/normal plasma cell ratio and lower expression of CD27, CD38, CD45, CD56, CD117 and CD138 in higher-risk genetic groups or risk categories.

## 1. Introduction

Multiple myeloma (MM) is an end point in the spectrum of plasma cell (PC) dyscrasias. In MM, malignant PCs (MMPCs) proliferate and damage target organs, such as bones, the bone marrow and kidneys [1]. At diagnosis, MM risk estimation includes factors such as patient performance status, disease burden, end-organ damage, biomarkers, and genetic abnormalities [2]. Increasing emphasis is applied on disease biology, specifically, genetic risk factors, which are especially important in patients who are young and in better health [3]. Traditional MM risk estimation methods such as the Durie–Salmon score were mostly based on disease burden. Later methods, such as the International Staging System (ISS), started to increasingly rely on biological factors. Current risk estimation methods, such as the revised-ISS (R-ISS) [4] and Stratification for Myeloma and Risk-Adapted Therapy (m-SMART) [5,6,7] include high-risk genetic abnormalities of MMPCs. Conventional karyotyping methods for detecting chromosomal abnormalities include G-banding and fluorescence in situ hybridization (FISH); however, Single Nucleotide Polymorphism (SNP) microarray molecular karyotyping allows far more detailed and precise chromosomal analysis. SNP microarrays have the benefit of detecting smaller changes (microdeletions and amplifications), hyperdiploidy or even more complex changes such as copy neutral loss of heterozygosity [8,9], especially, if plasma cells are separated prior to DNA extraction [10]. Methods such as gene-expression profiling (GEP) or next- generation sequencing (NGS) have been investigated; however, they have rarely been used in routine practice.

Multiparameter flow cytometry (MFC) is a method used in MM diagnostics. MFC enables the characterization of MMPC immunophenotype. It provides the basis for future minimal residual disease (MRD) testing [11] and helps to monitor the monoclonal gammopathy of undetermined significance (MGUS) progression to MM [12]. MFC is not always considered for MM risk estimation at diagnosis, due to the lack of independent marker significance [13]; however, MMPC immunophenotype has been shown to be related to prognosis [14,15,16] and some laboratory parameters [17]; it may also aid in predicting effectiveness of therapy [18,19]. MMPC immunophenotype may also indicate the maturity of cells and have some relation to genetic factors [20,21]. Detection of circulating plasma cells (CPCs) in peripheral blood (PB) by MFC at MM diagnosis can be used in the stratification of patient outcomes [22]. CPC numbers may also be estimated during treatment, to determine prognosis of the disease [23]. Next-generation flow (NGF) methods, such as MM-MRD panels and guidelines provided by the Euroflow consortium, significantly increase MM-MRD detection sensitivity and reliability. In addition, standardization of reagents and instrument settings makes the comparison of antigen expression profiles between laboratories easier.

We collected real-world data from newly diagnosed MM (NDMM) patients, on whose bone marrow a standardized NGF MM-MRD panel and detailed chromosomal analysis by SNP-A karyotyping and FISH was performed at diagnosis; we shall further describe the associations we discovered between the results of both testing methods.

## 2. Materials and Methods

We selected all NDMM patients admitted to Vilnius University Hospital Santaros Klinikos during 2019–2020, whose bone marrow was analyzed using a NGF MM-MRD assay, SNP-A karyotyping, and FISH. Inclusion criteria were age of 18 years or older, MM diagnosis based on IMWG criteria, no plasma cell leukemia diagnosis or active treatment for other cancers. The Durie–Salmon risk score, ISS stage, R-ISS stage and m-SMART risk score were calculated according to data available at diagnosis. R-ISS could not be calculated for two patients due to unavailable LDH measurements. Gene expression analysis (GEP) for high-risk signatures was not available for m-SMART stratification. High expression of ki-67 (>10%) by immunohistochemistry was used, whenever available, replacing high plasma cell S-phase markers in m-SMART criteria [24]. We modified the m-SMART categorization and labeled it m-SMART^A^.

CD138+ plasma cells were isolated using magnetic beads prior to SNP microarray karyotyping. All cases were investigated using the Infinium HD whole-genome genotyping assay with the HumanCytoSNP-12 BeadChip (Illumina Inc., San Diego, CA, USA), which covers the entire genome with an average spacing of 9.6 kb and allows an average resolution of 31 kb. Samples were processed and the assays were performed according to a routine protocol provided by the manufacturer. Genotypes were called by GenomeStudio GT module version 1.7 (Illumina Inc., San Diego, CA, USA), and further analyzed with QuantiSNP version 1.1 [25] and KaryoStudio version 1.0.3 software (Illumina Inc., San Diego, CA, USA). Constitutional copy number polymorphisms were excluded based on comparison with the Database of Genomic Variants (http://projects. tcag.ca/variation) (accessed on 15 March 2022).

All patients had MFC analysis performed from bone marrow aspirates, using the Euroflow instrument setting protocol for BD FACS Canto II (BD Biosciences, San Jose, CA, USA) flow cytometer, as well as the MM-MRD protocol for sample preparation (https://euroflow.org/protocols) (accessed on 2 January 2019). A Cytognos MM-MRD kit was used for sample staining. Spherotech (Lake Forest, IL, USA) SPHERO™ Rainbow Calibration Particles were run daily for performance tracking. Data acquired at diagnosis were analyzed using FACS Diva software (Becton Dickinson, San Jose, CA, USA). All PCs, including MMPCs and normal plasma cells (NPCs) were first discriminated from all other BM cells, with gates drawn generously to include as many MMPC and NPC events as possible. Lymphocytes, doublets, and other cellular debris were excluded through forward scatter and side scatter characteristics, while CD38, CD138 and CD45 expression was primarily used to select plasma cells, allowing the lower expression of these markers. MMPCs and NPCs were separated using the gate dot plots of two markers (CD27, CD81, CD117, CD19, CD45, CD56). The percentage of marker expression and intensity (as mean fluorescence intensity (MFI)) was recorded on both MM cell and normal PC cell populations (if detected above the lower limit of detection). Other cell populations were also gated, including NK/T cells (CD56+, SSC-low, CD45 + bright), B lymphocytes (CD19+, SSC-low, CD45 + bright), with CD27+ memory cell subpopulations separated. The level of detection for any cell population was more than 20 cells. Exact expression percentage was recorded for all markers, except for CD38 and CD138, based on which cells were gated generously to include events with dim expression of either marker. IBM SPSS Statistics 26 was used for statistical analysis. Marker expression and cell population percentages were tested for normality of data distribution. Since the distribution was not normal, non-parametric tests were performed—the Mann–Whitney U test for groups with two categories, the Kruskal–Wallis for multiple category comparison, with *p* < 0.05 chosen as the data significance level. Comparisons were made between groups with and without a genetic abnormality, unless specified otherwise. Survival analysis was performed using the Log Rank test and Kaplan Meier curves for different risk assessment categories, chromosomal aberrancies, and antigen expression (comparing patients with expressions above and below the group mean).

## 3. Results

We identified 73 patients that fitted inclusion criteria (Table 1). Since these were real world patients, they had worse Eastern Cooperative Oncology Group (ECOG) performance status than usually seen in clinical studies, and some would likely be excluded due to organ failure.

These were the patient treatment protocols: 35/73 (48%) received VTd (bortezomib, thalidomide and dexamethasone), 21/73 (29%) received CyBorD (cyclophosphamide, bortezomib and dexamethasone), 9/73 (12%) received IRd (ixazomib, lenalidomide and dexamethasone, 4/73 (5.5%) received D-VMP (daratumumab, bortezomib, melphalan and prednisone), 4/73 (5.5%) did not receive systemic treatment. A total of 32/73 (44%) received autologous stem cell transplantation (ASCT) in the course of their treatment.

**Figure 1:**
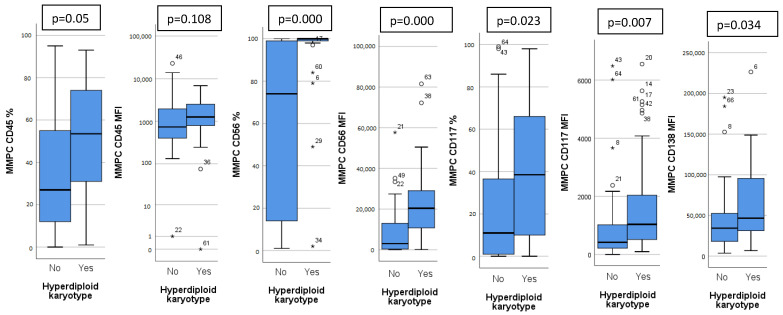
Significant differences in immunophenotype between hyperdiploid and non-hyperdiploid karyotype patients. CD45, CD56, CD117 measured as percentage of positive MMPC cells and mean fluorescence intensity (MFI), CD138 measured only as MFI. Outliers marked with *.

Patients were analyzed based on detected chromosomal abnormalities and immunoglobulin heavy chain (IgH) translocations. Thirty patients (41%) had a hyperdiploid karyotype (HK). Patients with HK had a distinct immunophenotype, with higher expression of CD45, CD56, CD117, CD138 on MMPCs and higher bone marrow MMPC percentage (Figure 1). Isolated HK (with no other chromosomal abnormalities) showed lower bone marrow MMPC percentage, lower MMPC/NPC ratio, when compared to non-isolated HK (Table 2).

High risk genetic abnormalities include t(4;14), t(14;16), t(14;20), 17 p deletion, p53 mutation and 1 q gain, as outlined in the m-SMART classification [7]. High risk genetic abnormalities were detected in 24 patients (32.9%). Sixteen patients (22%) had 1 q gain. There were no cases with 1 q amplification (>4 copies), associated with double hit MM [26]. Patients with 1 q gain showed higher CD27, CD117 expression than those without; higher MMPC/NPC ratio, lower NPC percentage. Eight patients (11%) had HK along with 1 q gain. When compared to non-HK patients with 1 q gain, they had a higher CD117 MFI and higher CD56+ T/NK cell percentage. Patients with t(4;14) (seven patients, 10%) had lower MMPC CD117 percentage and lower percentage of NPCs. Patients with 17 p deletion (nine patients, 12%) showed no significant differences in immunophenotype or cell percentages. t(14;16) and t(14;20) were detected in only one patient each, making data unsuitable for statistical analysis.

Higher disease risk may also be attributed to other genetic abnormalities, including chr 14 monosomy (14 q deletion), chr 13 monosomy and 1 p deletion. Eight patients (11%) had chr 14 monosomy or 14 q deletion, and showed lower MMPC CD117 expression and higher percentage of bone marrow MMPCs (Table 2). 23 patients (32%) had chr 13 monosomy. They had higher bone marrow MMPC percentage and higher MMPC/NPC ratio. Patients with 1 p deletion had lower CD27 expression on MMPCs, higher bone marrow MMPC and lower NPC percentage.

**Figure 2:**
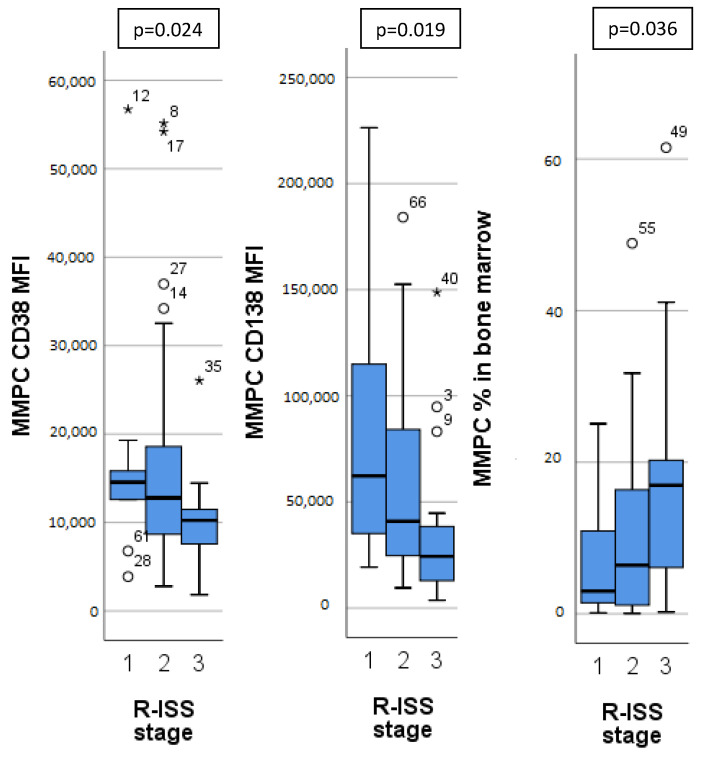
Significant differences in immunophenotype between different Revised—International Staging System (R-ISS) for multiple myeloma. MMPC—malignant plasma cells. MFI—mean fluorescence intensity. Outliers marked with *.

Different MM stages, as determined by ISS, R-ISS and m-SMART^A^ staging systems, had significant differences in MMPC immunophenotypes (Table 3). Patients with higher ISS and R-ISS stages had lower CD27, CD38 expression on MMPCs, higher bone marrow MMPC percentage, MMPC/NPC ratio, as well as lower hemoglobin concentration. Patients with higher R-ISS stages (Figure 2) additionally showed lower CD138 expression on MMPCs and lower B lymphocyte percentage. Similarly, m-SMART^A^ high risk category had lower CD27 expression on MMPCs, higher bone marrow MMPC percentage, lower hemoglobin concentration, and higher percentage of CD56+ T/NK cells in addition to that.

Copy number variation load (CNV load) was calculated in megabases, showing the magnitude of detected chromosomal abnormalities. It did not show a statistically significant relationship to immunophenotype, either in non-HK, or in HK patients. Most HK patients had some combination of chromosome 3, 5, 7, 9, 11 or 19 trisomy. The number or combination of duplicated chromosomes showed no specific relations to immunophenotype, except for the distinct HK immunophenotypic features already discussed.

Since a lot of comparisons were performed, we calculated Bonferroni corrected significance values (*p* < 0.003); however, the only significant differences with these criteria are CD56 expression (percentage and MFI) differences between HK and non-HK patients, as well as CD27 expression decrease (percentage and MFI) in ISS and R-ISS categories.

**Figure 3:**
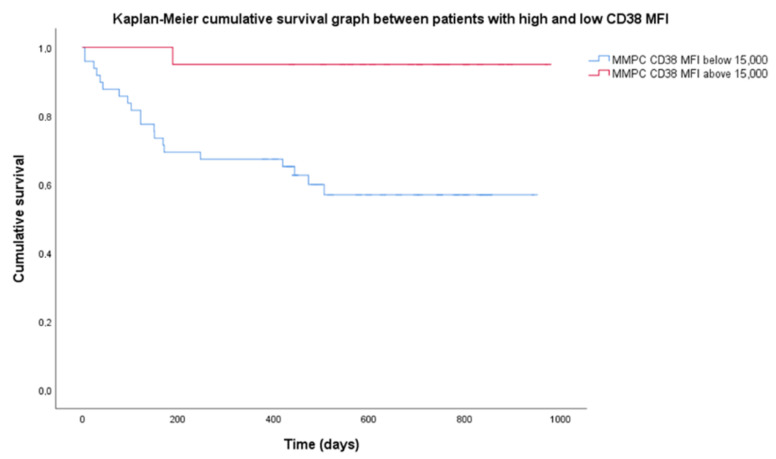
Difference in cumulative survival between subgroups of high and low CD38 expression. High expression of CD38 defined as mean fluorescence intensity (MFI) above 15,000 (20 patients), low—MFI below 15,000 (53 patients). MMPC—malignant plasma cells.

There were no statistical difference in survival in most patient categories since median survival was not reached. Median follow-up was 699 days. OS was 68.5% for the entire group. R-ISS stages showed different OS, with a significant log-rank test (*p* = 0.026). R-ISS stage 1 OS was 100%, stage 2–69%, stage 3–53%. Among chromosomal aberrancy groups, the most significant differences observed were isolated HK having a higher OS (90%); however, the log-rank test was not significant when OS was compared to non-isolated HK (60%). Higher CD38 expression resulted in prolonged OS. Mean CD38 MFI for the group was 14,700 (range 1830–56,727), so we chose 15,000 as the threshold for high CD38 expression. Twenty patients had high CD38 MFI while 53 patients had low CD38 MFI. Patients with low CD38 MFI had OS = 58.5%, when compared to high CD38 MFI with OS = 95%, and the Kaplan–Meier log-rank test was significant (*p* = 0.03) (Figure 3).

## 4. Discussion

Chromosomal abnormalities are becoming increasingly relevant in MM prognostics, and methods such as SNP-A karyotyping enable thorough and reliable analysis of chromosomal changes. Combinations of high and standard risk abnormalities provide a complex picture that could make clear prognosis difficult. MMPC antigen expression may provide additional information in risk assessment. We found specific differences in immunophenotype when comparing different genetic risk factors. Specifically, lower expression of CD27, CD38, CD45, CD56, CD117 and CD138, as well as higher MMPC/NPC ratio showed lower expression on higher risk genetic groups or risk categories. Studies have shown immunophenotype that plays a role in MM risk assessment, such as CD19pos, CD27neg, CD38lo, CD45pos, CD81pos, CD117neg and CD138lo as separate markers, or CD38low, CD81pos, CD117neg as a combination in a study by Arana et al. [16].

Tarín et al. [13] showed that relapsed/refractory MM (RRMM) patients exhibit lower CD27 and higher CD81 expression, while Pojero et al. [27] showed that progressive MM has lower CD27 expression. CD27 is a tumor necrosis factor receptor highly expressed on plasma cells, whose loss has been associated with poor prognosis [28]. Arana et al. [16] found that negative CD27 and positive CD81 expression is related to poor outcomes. We found CD27 downregulation in higher risk categories such as 1 q gain, 1 p deletion, as well as CD27 decrease with higher ISS, R-ISS stages and m-SMART^A^ categories (Table 3), all of which relate to higher risk. We found no CD81 expression differences in any category.

Nijhof et al. [19] showed that patients who achieved partial response (PR) after daratumumab therapy had significantly higher CD38 expression than those that did not achieve at least PR. CD38 is a protein that has a role in cell adhesion, expressed in very high levels by NPCs and MMPCs, and has been used as a target for monoclonal antibody therapy [29]. Our patients were evaluated at diagnosis, so the differences in expression were present before therapy. Additionally, access to daratumumab was limited for these patients, and only four (5.5%) received anti-CD38 treatment and were excluded from OS analysis. Since targeted anti-CD38 therapy was not used, lower OS in patients with CD38 MFI below 15,000 (Figure 3) may be related to prognostic significance of CD38 expression itself. Arana et al. [16] showed that low CD38 expression is part of the immunophenotype with the highest risk.

We found higher CD45 expression on MMPCs of patients with HK (Figure 1), who tend to have better outcomes. In some studies, lower CD45 expression on MMPCs has been associated with worse outcomes [30]. However, Arana et al. [16] described positive CD45 expression as predictive of inferior outcomes, similarly to Gonsalves et al. [31]. Our sample size was too small for definitive determination of these inconsistencies. In general, MMPCs of patients with HK showed a significantly different phenotype compared to non-HK patients. While CD45 expression differed slightly, CD117 and CD138 showed significant differences, and CD56 expression differed greatly (Figure 1). Some differences in immunophenotype have been described previously [32] with MMPCs of HK patients having higher expression of CD56, CD117, as well as lower expression of CD20 and CD28. Lower CD56 expression has been linked to lower effectiveness of Bortezomib [18], so higher expression may have a beneficial impact on outcome. CD117 was more highly expressed by HK patient MMPCs; however, it also showed difference in other patient groups (Figure 1). CD117 was lower in patients with 1 q gain, t(4;14) and chr 14 monosomy/14 q deletion (Table 2). Chr 14 monosomy or 14 q deletion are not always included in risk estimation; however, chromosome 14 includes the IgH gene, which is involved in translocations relevant to MM pathogenesis [1]. Guo et al. [15] showed that lower MMPC CD117 expression, as well as higher percentage of bone marrow MMPCs were related to shorter PFS and OS; they also noted the relationship between CD117 and chromosomal abnormalities—17 p deletion, 1 q gain and IgH translocations. We found no specific immunophenotypic features in patients with 17 p deletion (9 patients, 12%). CD117 negative expression was noted as part of the most predictive for poor outcomes by Arana et al. [16]. CD138 had higher expression on HK patient MMPCs, and lower expression by patients with higher R-ISS stages (Figure 2) and ISS stages (Table 3). Arana et al. [16] described CD138 low expression as predictive of inferior outcomes. Lower CD56 and CD138 expression has also been linked to the presence of CPCs in PB, which is related to disease progression [33].

Decrease in NPC percentage at MM diagnosis, when compared to healthy donors, has been described previously [34], and has been linked to competition for the same bone marrow niche [35]. Higher MMPC percentage in the PC compartment has been linked to progression from SMM to MM [36]. We found different risk categories and genetic abnormalities related to high risk had higher bone marrow MMPC and/or lower NPC percentage (non-isolated HK, chr 14 monosomy/14 q deletion, chr 13 monosomy, 1 p deletion, as well as ISS, R-ISS and m-SMART^A^ risk categories. Patients with 1 q gain and t(4;14) had decreased bone marrow NPC percentage, without related MMPC percentage increase (Table 2). It is not clear whether these changes in the PC compartment depend on tumor burden or show the effect of progressive disease on the bone marrow.

## 5. Conclusions

We detected specific MMPC immunophenotype features in patients with different MM risk factors at diagnosis, as determined by current risk stratification methods or detection of specific genetic abnormalities. Risk stratification methods that do not involve genetics, such as ISS, also showed specific trends in MMPC immunophenotype. Lower expression of CD27, CD38, CD56, CD117 and CD138, higher bone marrow MMPC and lower bone marrow NPC percentage, determined at diagnosis by MFC, were related to factors that determine higher-risk disease. Some chromosomal changes (such as CNV load or 17 p deletion) had no specific immunophenotypic features, as well as some markers (CD81, CD45) not showing specific differences in relation to genetics. There was immunophenotypic overlap between different high-risk chromosomal abnormalities, so it may be possible that a specific combination of markers shows increased disease risk and would benefit from more intense treatment strategies. However, larger-scale long-term studies employing standardized MFC analysis at diagnosis would help to determine the precise importance of these factors in patient survival.

## Figures and Tables

**Table 1:** Patient characteristics with categorical variables as percentages and continuous variable mean and range values.

Characteristic	Number of Patients (%) Total *n* = 73
Male	30 (41.1%)
Female	43 (58.9%)
R-ISS stage 1	10 (13.7%)
R-ISS stage 2	42 (57.5%)
R-ISS stage 3	19 (26.0%)
m-SMART^A^ * standard risk	35 (47.9%)
m-SMART^A^ high risk	38 (52.1%)
Normal karyotype	19 (26.0%)
Standard risk abnormalities	30 (41.1%)
1 high risk ** abnormality	21 (28.8%)
2 high risk abnormalities	3 (4.1%)
IgG	36 (49.3%)
IgA	15 (20.5%)
Unknown or light-chain only	22 (30.1%)
	Mean (range)
Age at diagnosis	67 (42–84)
ECOG/WHO performance status	1 (0–4)
HGB (g/L)	110 (67–185)
Creatinine	92 (38–920)
Beta-2-microglobulin	4.9 (1.38–47.41)
Albumin	40 (15.9–54.7)
LDH	199 (104–697)
Ca^2+^	1.22 (1.04–2.61)

* Modified m-SMART categorization—gene expression analysis (GEP) was not available, high ki-67 (>10%) by immunohistochemistry was used when available replacing high plasma cell S-phase. ** High risk genetic abnormalities include t(4;14), t(14;16), t(14;20), 17 p deletion, p53 mutation and 1 q gain.

**Table 2:** Differences in immunophenotype (by MFI or percentage median), bone marrow cell percentages and laboratory test values between different genetic MMPC subgroups. MFI—mean fluorescence intensity, MMPC—malignant plasma cells, NPC—normal plasma cells. Statistically significant (*p* < 0.05) differences in bold and marked with *; arrow indicates if value is higher or lower than the contrary subgroup.

Marker Expression (%, MFI), Median (total = 73 patients)	Hyperdiploidy (Non-Isolated) 20 (27%)	Hyperdiploidy (Isolated) 10 (14%)	1q Gain Detected 16 (22%)	1q Gain not Detected 57 (78%)	t(4;14) Detected 7 (10%)	t(4;14) not Detected 66 (90%)	Chromosome 14 Deletion Detected 8 (11%)	Chromosome 14 Deletion not Detected 65 (89%)	1p Deletion Detected 9 (12%)	del1p not Detected 64 (88%)
**CD27 %**	64	82	62	77	56	77	69.5	74	43 *↓	78 *↑
**CD27 MFI**	1313	2523	1118 *↓	2532 *↑	1123	2442	2624	2323	1123 *↓	2619 *↑
**CD38 MFI**	11,146	13,033	10,679	12594	12,006	11,848	10,726	12,006	10,965	11,945
**CD45 %**	48.5	62.5	32	42	30	41	31.5	41.5	42	40.5
**CD45 MFI**	1162	1524	1089	900	1089	908	865	919	916	911
**CD56 %**	100	100	100	98	100	97	95	99	100	97.5
**CD56 MFI**	19,210	21,590	11,271	9452	11,645	8885	6634	11,314	11,271	9966
**CD117 %**	31	61	2 *↓	20 *↑	1 *↓	21 *↑	1 *↓	19 *↑	4	21
**CD117 MFI**	929	1494	326 *↓	657 *↑	225	649	215 *↓	632 *↑	408	649
**CD138 MFI**	45,732	47,978	39,489	38436	31,296	39,003	34,173	39,321	37,720	39,338
**MMPC % in BM**	16.7 *↑	4.1 *↓	7.3	8,4	6.1	7.0	21.8 *↑	7.2 *↓	20.5 *↑	6.0 *↓
**NPC % in BM**	0.026 *↓	0.068 *↑	0.013 *↓	0.033 *↑	0.012 *↓	0.033 *↑	0.012	0.031	0.012 *↓	0.035 *↑
**MMPC/NPC ratio**	538.2 *↑	71.3 *↓	566.3 *↑	168.5 *↓	317.0	189.0	1145.7 *↑	168.5 *↓	1938.6 *↑	157.7 *↓
**CD56+ T/NK cell %**	29.9 *↑	21.4 *↓	25.3	24,6	26.3	24.6	26.8	24.5	32.9 *↑	24.3 *↓
**CD27+ T cells percentage**	40 *↓	47 *↑	41	44	41.3	43.9	43.7	43.8	41.3	44.1
**B lymph percentage**	6.8	11.7	6.8	9,4	11.4	8.6	4.7*↓	9.4*↑	5.6	9.1
**Beta-2-microglobulin**	6.4 *↑	3.3 *↓	5.8	4,5	5.7	4.7	7.4	4.9	14.3 *↑	4.1 *↓
**Hemoglobin (g/L)**	97.5 *↓	123 *↑	96.5 *↓	115 *↑	97 *↓	113 *↑	100.5	110	98 *↓	113 *↑

**Table 3:** Differences in immunophenotype (by MFI or percentage median), cell percentages and laboratory test values between different risk assessment stages. MFI—mean fluorescence intensity, MMPC—malignant plasma cells, NPC—normal plasma cells. Statistically significant (*p* < 0.05) differences marked with *; arrow indicates if value is higher or lower than the contrary subgroup.

Marker Expression (%, MFI), Median (Total = 73 Patients)	MSMART (2007) High Risk 35 (48%)	MSMART (2007) Standard Risk 38 (52%)	R-ISS Stage			ISS stage		
			1 10 (14%)	2 42 (58%)	3 19 (26%)	1 25 (34%)	2 16 (22%)	3 30 (41%)
**CD27 %**	64 *↓	85 *↑	86 *↑	78 *↑	55 *↓	74 *↑	94 *↑	57 *↓
**CD27 MFI**	2017	2626	2442 *↑	2813 *↑	1103 *↓	2531 *↑	3897 *↑	1373 *↓
**CD38 MFI**	11,667	11,884	14,560 *↑	12,783 *↑↓	10,238 *↓	14,895 *↑	12,440 *↓↑	10,106 *↓
**CD45 %**	34	44	36	44	37	36	38	45
**CD45 MFI**	919	900	794	1129	919	871	972	1006
**CD56 %**	98.5	98	99	97	99.5	99	93	99
**CD56 MFI**	10,097	11,691	15,159	6622	11,050	11,691	9903	9017
**CD117 %**	13	32	41	6	22	23	4	18
**CD117 MFI**	451	781	1111	411	691	693	291	613
**CD138 MFI**	37,536	41,261	36,217 *↑	41,261 *↓↑	27,662 *↓	40,490	38,792	32,820
**MMPC % in BM**	8.4 *↑	2.5 *↓	3.5 *↓	6.8 *↑	14.4 *↑	3.5 *↓	2.6 *↓	16.7 *↑
**NPC % in BM**	0.022 *↓	0.044 *↑	0.041 *↑	0.034 *↓↑	0.013 *↓	0.042	0.032	0.022
**MMPC/NPC ratio**	317 *↑	88.1 *↓	42 *↓	169 *↑	501 *↑	65.7 *↓	140.6 *↓	435.2 *↑
**CD56+ T/NK cell percentage**	26 *↑	23.5 *↓	28.8	24	30	25.9	22.9	28.0
**B lymph percentage**	6.7	9.7	13.8 *↑	9.4 *↓↑	5.5 *↓	9.9	7.4	5.9
**Beta-2-microglobulin**	5.8	3.7						
**Hemoglobin (g/L)**	99.5 *↓	116 *↑	119.5 *↑	113 *↓↑	94 *↓	124 *↑	118 *↓↑	97 *↓
**Albumin**	40.1	39.7						

## Data Availability

Data not shared due to privacy restrictions.
